# Yang’s Classification of Juvenile TMJ Anterior Disc Displacement Contributing to Treatment protocols

**DOI:** 10.1038/s41598-019-42081-5

**Published:** 2019-04-04

**Authors:** Pei Shen, Qianyang Xie, Zhigui Ma, Ahmed Abdelrehem, Shanyong Zhang, Chi Yang

**Affiliations:** 1 Department of Oral Surgery, Ninth People’s Hospital, College of Stomatology, Shanghai Jiao Tong University School of Medicine, Shanghai Key Laboratory of Stomatology & Shanghai Research Institute of Stomatology, Shanghai, People’s Republic of China; 2 Department of Craniomaxillofacial and Plastic Surgery, Faculty of Dentistry, Alexandria University, Alexandria, Egypt

## Abstract

This study aims to establish a new staging system of temporomandibular joint (TMJ) anterior disc displacement (ADD) and evaluate its role in guiding the treatment plan. A consecutive sample of 522 juvenile patients (780 joints) diagnosed as ADD based on magnetic resonance imaging (MRI) was included and analyzed. 674 joints received TMJ treatments according to our staging system, while 106 joints rejected any treatment; only for follow-up. The outcomes were judged according to our success criteria. The prognosis of our staging system was also evaluated in comparison to Wilkes classification. Kaplan–Meier survival analysis showed that significant stratifications of the ameliorative rate were found at all subgroups within any two stages in our staging system, except for subgroups between stages 0 and 1, stages 2 and 3, and stages 3 and 4. After analyzing the interactions between different candidate prognostic factors in a Cox model, the relative risks of deterioration of ADD included treatment methods (HR = 42.94, P < 0.0001), disease course (HR = 0.98, P = 0.0019), stages of ADD (HR = 3.81, 9.62, 12.14, P = 0.016, 0.000,0.000 respectively for stage 2, stage 3 and stage 4) and the interaction between ADD stages and treatment methods. The C index of this model was 0.87. The new staging system of TMJ ADD appears reliable, and benefits to making treatment planning and predicting the prognosis.

## Introduction

Temporomandibular joint (TMJ) anterior disc displacement (ADD) is one of the most common TMJ disorders. It can occur in all age groups, with a high prevalence in adolescents^1,2^. ADD often presents with clicking, joint pain, a limited range of mouth opening, and masticatory difficulty. Furthermore, ADD might lead to osteoarthrosis and decrease in condylar height. In recent years, its correlation with mandibular growth has drawn more and more attention^3,4^. Back to 1966, Boering^5^ first reported mandibular growth disturbance in the young patients with signs and symptoms of TMJ osteoarthrosis and internal derangement. Thereafter, more studies demonstrated that a significant mandibular deficiency and malocclusion could develop in patients with condylar deformity and ADD^6,7^. In the last 20 years, many orthodontists have related ADD in growing patients with the decreased ramus height and mandibular body length^8–11^. Moreover, these results were also confirmed in animal models. ADD model in growing rabbits showed that the surgically created unilateral ADD could result in shortening of the mandibular ramus and mandibular asymmetry, while bilateral TMJ disc displacement could induce mandibular retrognathia^12–15^. All these studies suggested that ADD might be one of the causative factors for dentofacial deformities, especially for facial asymmetry and mandibular retrognathia.

Since ADD of the TMJ can lead to various harmful outcomes, how to manage ADD is considered a key problem for most TMJ experts. In the last 20 years, disc reposition, as being a conventional treatment for ADD patients, has long gained a wide recognition^16–19^. On the other hand, TMJ replacement has been introduced for clinical application on those whose TMJ conditions are serious^20,21^. Many studies reported that correcting the disc position by suitable repositioning methods can help preventing condyle resorption or even can correct dentofacial deformities^22,23^. Nevertheless it has been difficult for most clinicians to select a suitable method for ADD patients with different grades of severities.

Actually there were no ideal classification evaluating the severity of ADD so far, in addition to considering the criteria for the selection of treatment protocols. Although Wilkes has provided a staging criteria for internal derangements (ID) of the TMJ based on the clinical, radiological, and surgical findings^24^, this did not pay a special attention to the relationship between disc displacement and condyle bony changes and resorption. Besides Wilkes did not propose the treatment criteria for patients with different stages. Therefore, the aim of the current study was to establish a new staging system of ADD and evaluate its role in guiding the treatment plan. The prognosis of the new classification was also compared with Wilkes staging system in this study for reliability and validity purposes.

## Results

### Patients Basic Data

The study population included 522 patients (396 females and 126 males) with a total of 780 joints. The mean age at their first visit was 15.8 ± 3.6 years old and the average disease course was 25.6 ± 15.5 months. The basic demographics of the ADD patient population as well as Wilkes staging and our new staging distributions were presented in Table 1. Among the total 780 joints, 674 joints underwent treatment according to our staging system, while the other 106 joints rejected any treatment. The average follow-up period was 20 months (range 1 to 86 months).

**Table 1:** Descriptive Statistics of the Patients.

Variable	Value	Percentage
Age	15.8 ± 3.6	
Gender		
Male	126	
Female	396	
joints	780	
Disease course(Mo)	25.6 ± 15.5	
Our staging		
0	253	32.44%
1	223	28.59%
2	111	14.23%
3A	120	15.38%
3B	25	3.21%
4A	28	3.59%
4B	20	2.56%
Wilkes staging		
1	27	3.46%
2	108	13.85%
3	303	38.85%
4	249	31.92%
5	93	11.92%
Treatment method		
Yes	674	86.41%
No	106	13.59%
Follow-up time	20.0 ± 16.8	

### Staging Systems and Ameliorative rate

**Figure 1:**
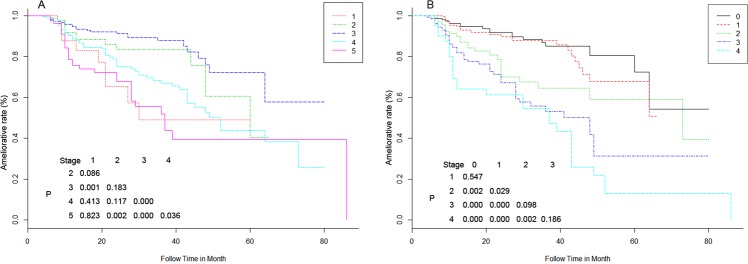
Kaplan–Meier survival curves by patient subtype with different staging systems. Overall ameliorative curves of subgroups of patients with both staging systems (X: Ameliorative rate (%); Y: Follow time in month). (**A**) The ameliorative curves of subgroups of patients with Wilkes staging. (**B**) The ameliorative curves of subgroups of patients with our staging system.

First, the proposed new staging and Wilkes staging systems were independently analyzed using the Kaplan–Meier survival analysis (Fig. 1). The differences between Wilkes stages 1 and 2 (x^2^ = 3, P = 0.086), stages 1 and 4 (x^2^ = 0.7, P = 0.413), stages 1 and 5 (x^2^ = 0.1, P = 0.823), stages 2 and 3 (x^2^ = 1.8, P = 0.183), and stages 2 and 4 (x^2^ = 2.5, P = 0.117) were not statistically significant. However the differences in Wilkes staging system between stages 1 and 3 (x^2^ = 11.3, P = 0.001), stages 2 and 5 (x^2^ = 9.5, P = 0.002), stages 3 and 4 (x^2^ = 16.5, P = 0.000), stages 3 and 5 (x^2^ = 32.5, P = 0.000), and stages 4 and 5 (x^2^ = 4.4, P = 0.036) were statistically significant. In our staging system, significant stratifications of the ameliorative rate were found at all subgroups within any two stages, except for subgroups between stage 0 and stage 1 (x^2^ = 0.4, P = 0.547), stage 2 and stage 3 (x^2^ = 2.7, P = 0.098), as well as stage 3 and stage 4 (x^2^ = 1.8, P = 0.186). These results implied that our staging system manifested a significant stratifications in different subgroups compared with Wilkes staging system.

### Univariate Cox Regression Analysis

Then the impact that various factors had on the prognosis using a univariate Cox regression analysis, was investigated (Table 2). Among all the variables, age, our stage (2, 3, 4), Wilkes stage 2 and the treatment method were significantly associated with the success rate (p < 0.05, respectively). Comparing both staging systems, our staging system showed much better predictability of the outcomes.

**Table 2:** Univariate and Multivariate Analyses for Deterioration of ADD Patients.

variables	Univariate Analysis		Multivariate Analysis	
	Hazard ratio (95%CI)	P	Hazard ratio (95%CI)	P
gender - M vs F	0.66 (0.41–1.05)	0.0809		
Age	1.22 (1.11–1.34)	<0.0001		
Disease course	1.02 (0.75–1.37)	0.9226	0.98 (0.97–0.99)	0.0019
Wilkes staging				
1 vs 3	3.44 (1.61–7.34)	0.0703		
2 vs 3	1.59 (0.83–3.04)	0.0014		
4 vs 3	2.56 (1.61–4.07)	0.4139		
5 vs 3	4.11 (2.44–6.94)	0.6359		
Our staging				
1 vs 0	1.22 (0.67–2.25)	0.5121	1.65 (0.54–5.05)	0.3791
2 vs 0	2.39 (1.32–4.33)	0.0042	3.81 (1.32–4.33)	0.0167
3 vs 0	3.97 (2.34–6.73)	<0.0001	9.62 (3.69–25.08)	<0.0001
4 vs 0	5.55 (3.11–9.92)	<0.0001	12.14 (4.09–36.01)	<0.0001
Treatment				
(no vs yes)	13.25 (9.39–18.70)	<0.0001	42.94 (15.38–119.92)	<0.0001
Our stage 1 * treatment(yes)			0.86(0.23–3.30)	0.83
Our stage 2 * treatment(yes)			0.3(0.08–1.11)	0.0713
Our stage 3 * treatment(yes)			0.19(0.06–0.61)	0.0053
Our stage 4 * treatment(yes)			0.14(0.04–0.49)	0.0024

### Multivariate Cox Regression Analysis and Covariate Interactions

Next, we compared all combinations of candidate prognostic variables. The multivariate Cox proportional hazards model identified several important predictors of outcomes (Table 2): treatment, disease course and our staging system. The Hazard ratios of the treatment and disease course were 0.98 (P = 0.0019) and 42.94(P < 0.0001) respectively. Hazard ratios of our stage 1, 2, 3 and 4 versus stage 0 were 1.65(CI:0.54–5.05, P = 0.3791), 3.81(CI:1.32–4.33, P = 0.0167), 9.62(CI: 3.69–25.08, P < 0.0001) and 12.14(CI:4.09–36.01, P < 0.0001) respectively, which suggested that the prognosis became worse as the stage increased. Interaction between covariates was also analyzed in the multivariable model. The stratification by our staging system demonstrated a significant interaction with the treatment methods. The hazard ratios for our stages 1, 2, 3, and 4 interacted with the treatment methods were 0.86(P = 0.83), 0.3(P = 0.0713), 0.19(P = 0.0713) and 0.14(P = 0.0024) respectively.

### Validation of the Predicting Model

**Figure 2:**
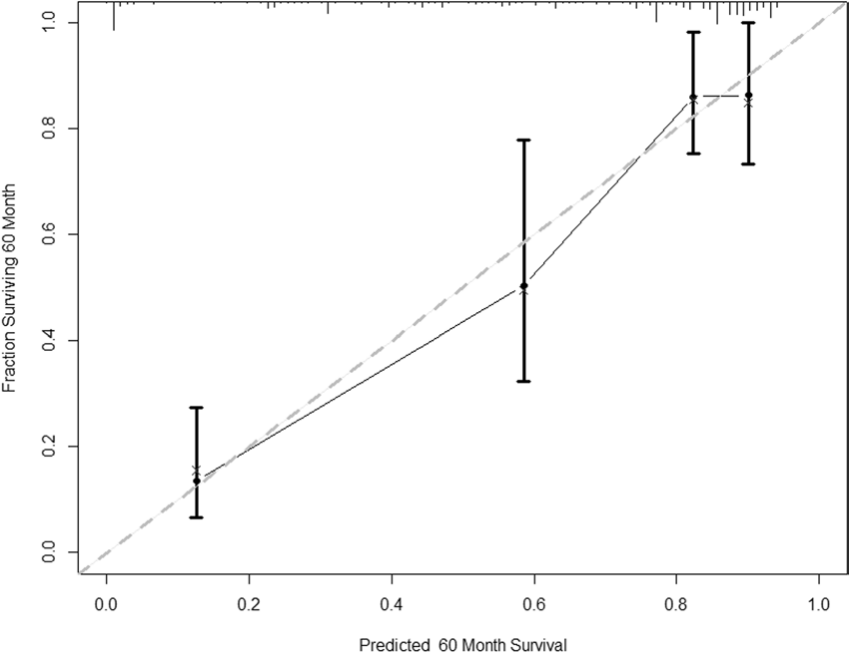
Nomogram (Cox model) Predicted 60 months deteriorative probability. Bootstrap estimate of calibration accuracy for five-year(60 months) estimates from the final Cox model. Dots correspond to apparent predictive accuracy. X marks the bootstrap-corrected estimates.

Validation of the predicting model on our dataset was performed at last. The C index of 0.87 indicated 87.0% accuracy in ranking our patients according to the risk of deterioration. Calibration was assessed at 60 months from their first visit to allow a direct comparison with our models. The calibration plot of the predicted versus observed probabilities of amelioration showed good calibration with an accurate risk estimation (Fig. 2).

## Discussion

Condylar resorption of the TMJ, which predominately occurs in adolescents, can lead to mandibular asymmetry or retrognathia and other dentofacial deformities^25,26^. Despite a number of theories, the causation of condylar resorption has never been exactly demonstrated so far.

ADD, as one of the most common TMJ diseases, has gained an extensive attention not only in the TMJ field but also in the orthodontics and orthognathics fields. The reason might be the potential correlation between ADD and condylar resorption. Many studies have reported that ADD in teenagers is closely related to mandibular asymmetry and mandibular deficiency, particularly retrognathia^27,28^. Orthognathic surgeons have found that 88% of patients who had an orthognathic surgery for a class II malocclusion had TMJ disc displacement^29^. Moreover, surgeons have noted an increased rate of Class II relapse following orthognathic surgeries in young patients with ADD^30,31^. In our clinic, we also found that patients with ADD often had condylar resorption of the TMJ. Besides, the longer ADD history, the more severe condyle resorption and dentofacial deformities^32,33^. All of the above-mentioned results indicate that juvenile ADD is a major causative factor for condylar resorption resulting in mandibular asymmetry and retrognathia.

Although the potential relationship between ADD and condylar resorption has been implied in many studies^34,35^, there are no reports describing the severity of this disease. In the present study, we have designed a new staging system for juvenile ADD and condylar resorption based on MRI images. Since MRI is a noninvasive technique which allows a valid and reproducible assessment for both osseous components and soft tissues of the TMJ, it is used as a primary evaluation method for staging in the present study. The superior imaging sensitivity of MRI allows for detecting cartilage and bony changes at an early stage. In our staging system, we have divided juvenile ADD into five stages according to the degrees of condylar resorption and status of the disc, as well as considering stages 3 and 4 as ADD with severe condylar resorption. The last two stages have been further subdivided into sub-stages A and B, based on the shape and length of the disc, inflammatory changes in the condyle, and the presence of bone marrow in the condylar head. The purpose of such sub-staging is to reflect the ability of condylar regeneration and possibility of disc repositioning. The authors believe that stages 3 and 4 juvenile ADD actually represents what has previously been termed idiopathic condylar resorption (ICR).

Another aim of our staging system is to provide a reasonable treatment protocol for ADD patients with varying severities. Since the length of the disc in stages 0, 1, 2 and 3A is barely enough to cover the top of the condyle, reposition treatments, including functional splint and arthroscopic or open surgeries, are recommended. Actually by many years follow-up, we found that a quite number of juvenile ADD patients had condylar regeneration with new bone formation after disc reposition, which will be presented in our future article. As for stage 3B patients, because of their markedly distorted disc and seriously resorbed condyle, disc reposition is deemed as noneffective. If these patients lacked any TMJ symptoms with a disc–like character in the TMJ bilaminar zone, then MRI was repeated after 6 to 12 months to determine whether condylar resorption had stopped. Once this was ascertained, the dentofacial deformity was corrected using moderate orthodontics and/or orthognathic surgery but without TMJ treatment. If condylar resorption continued, the patient was then treated according to stage 4. In this stage, since condylar bone marrow is markedly reduced (stage 4A) or absent (stage 4B), we believed that little or even no condylar regeneration would be possible. Thus, in this case, we routinely resect the condyle and replace it with costochondral grafts (CCG) or total alloplastic TMJ replacements.

To evaluate our staging system, we compared it with Wilkes’s classification. Although Wilkes staging system was widely used in the TMJ field, its predictive value for the TMJ is still unclear. In the present study, all the cases were classified according to both staging systems. By using the Kaplan–Meier survival analysis, significant stratifications in our staging system were observed compared with Wilkes staging system. This implied that our system is superior to Wilkes system in the prognostic value of the disc displacement.

To find which variables were related to the prognosis, we have also used Cox regression analysis. Our results showed that the treatment methods, disease course, our stages and the interaction of our stages and treatment methods were associated with the prognosis. The higher stage and absence of treatment method were especially detrimental and correlated with the deterioration. Although the disease course was a risk factor for the deterioration by the univariate analysis, it became protective in the multivariate Cox model. We think this is because the disease course is relevant to our stage. That means the longer disease course, the higher ADD stage^33^. However when ADD patients were processed by suitable treatments, their prognosis became reversed. The predictive accuracy and calibration manifested that this model had a good predictive value.

To our knowledge, the ADD staging system is the first to demonstrate the severity of TMJ disc displacement and its relationship with condylar resorption. The results have shown that this staging system is feasible, and benefits in guiding the treatment protocol and predicting the prognosis.

However we still can not elaborate why ADD in young patients will deteriorate into serious condylar resorption, and its pathogenic mechanism needs to be further explored in the future. Certainly, a longitudinal study of ADD will give us more information regarding this. From our results, we think that it is very important to inhibit or reverse the deteriorating changes of the condyle secondary to ADD at an early stage. We emphasize that the salvageable disc should be repositioned as early as possible.

## Conclusion

In conclusion, we believe that the new staging system of TMJ ADD appears reliable, and benefits in guiding the treatment plan and predicting the prognosis.

## Patients and Methods

### Ethics Statement

This study followed all the tenets of the Declaration of Helsinki for research involving human subjects, and was critically reviewed and approved by the institutional review board of Shanghai Jiao Tong University School of Medicine. An informed written consent was obtained from all participants or their legal guardian (patients under the age of 18 years).

### Patients Selection

A total of 3177 consecutive juvenile patients (age range from 10 to 20 years) who visited the TMJ Clinic at the Department of Oral Surgery in Shanghai Ninth People’s Hospital from August 2009 to October 2012 were reviewed in the current study. Among them, those who were diagnosed as ADD of the TMJ based on magnetic resonance imaging (MRI) scanning according to the reported methods^36,37^ and had more than one time follow-up MRI scanning in our department, were included in our study. The patients with histories of congenital deformities (e.g. microsomia, Treacher Collins syndrome), systemic disease (e.g. juvenile rheumatoid arthritis), infection, jaw fracture, or other clinically significant pathologies affecting the growth of the condylar were excluded in our study. Demographics and clinical course were collected from the recorded data of all included cases. The duration between first occurrence of clicking, pain of the TMJ or mouth opening limitation and their first visit to our department was considered as the disease course.

### The new staging system for ADD patients

**Figure 3:**
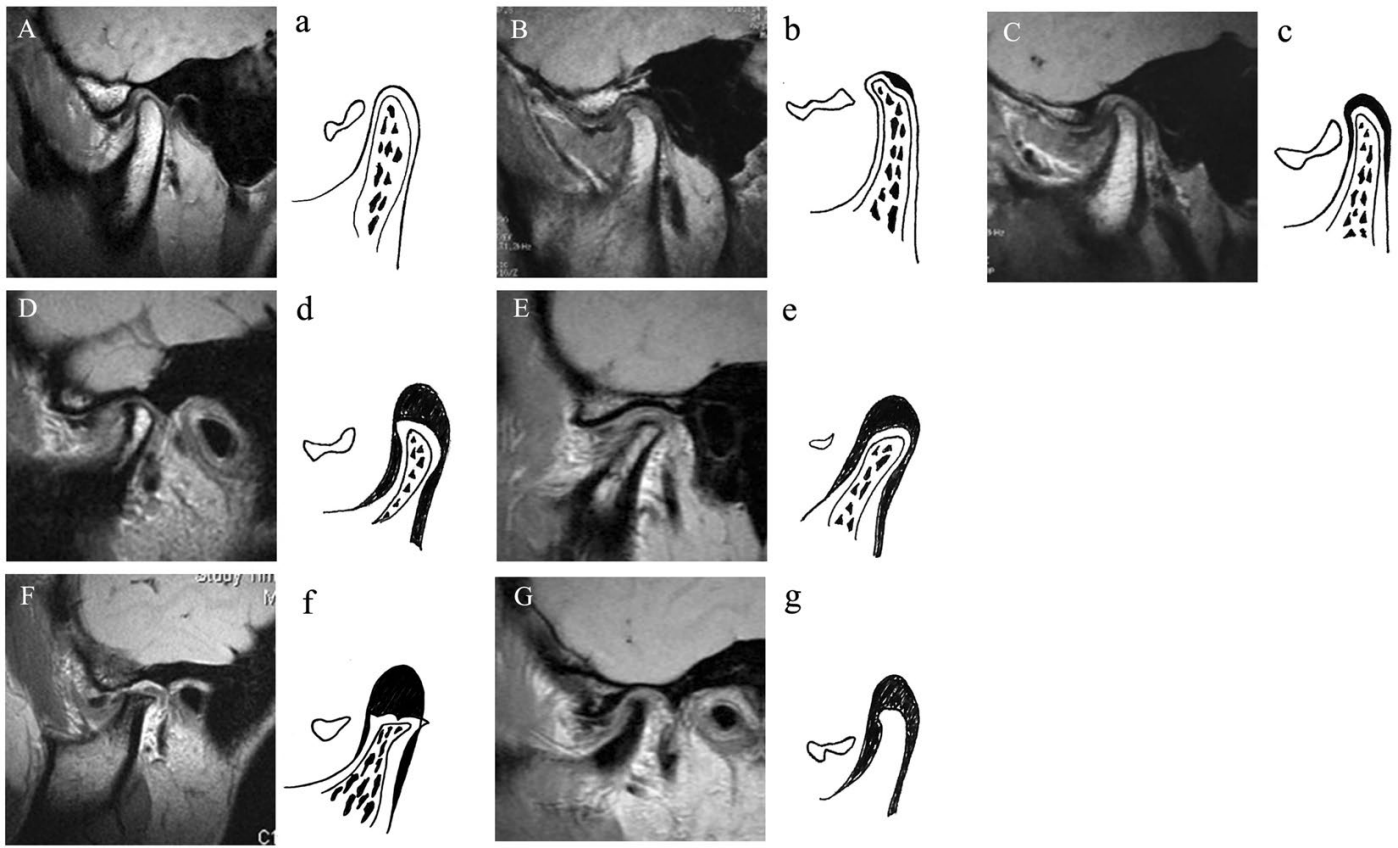
Juvenile TMJ anterior disc displacement staging system.

Primarily based on MRI, juvenile ADD is divided into 5 stages according to the degree of condylar resorption and status of the disc. ***Stage 0:*** MRI shows basic shape of the disc with normal condylar shape and height, also the marrow of the subchondral bone has normal volume and quality. ***Stage 1:*** the disc has a basic shape and the condyle shows mild or local resorption, however, still with normal height. In addition, the marrow of the subchondral bone is partially reduced on the top. ***Stage 2:*** the disc has a basic shape and the condyle appears as moderately resorbed with reduced height, while the marrow of the subchondral bone becomes mildly reduced. ***Stage 3:*** the shape of disc is basic or distorted and the condyle has severe resorption. Also the marrow of the subchondral bone is moderately reduced. ***Stage 4:*** the conditions of disc and condyle are the same as stage 3, but the marrow of the subchondral bone is moderately reduced with inflammatory changes, severely reduced, or even totally absent (Table 3, Fig. 3). According to the shape and length of the disc, inflammatory changes in the condyle, and the presence of bone marrow in the condylar head, stage 3 and stage 4 were subdivided into sub-stages A and B (Table 3, Fig. 3).

**Table 3:** Stage for Juvenile TMJ Disc Displacement Based on MRI.

stage	Disc	Condyle	Marrow
Stage 0	Basic shape	Normal condylar shape and height	Normal volume and quality
Stage 1	Basic shape	Mild and local condylar resorption, but normal condylar height	Partially reduced on the top
Stage 2	Basic shape	Moderate condylar resorption, reduced condylar height.	Mildly reduced
Stage 3	Basic shape or distorted	Severe condylar resorption	Moderately reduced
3A	Basic shape remains, or mildly distorted and shortened	Small, but basic shape is present	Moderately reduced
3B	Severely distorted and shortened	Small, but basic shape is present	Moderately reduced
Stage 4	Basic shape or distorted	Severe condylar resorption	Moderately reduced with inflammatory changes, or severe reduced, or absent
4A	Basic shape remains or distorted. Perforation is common	Severe resorption, loss of integrity of cortical bone.	Moderately reduced with severe inflammatory changes
4B	Basic shape remains or distorted. Perforation is common	Severe resorption, or complete resorption	Severe reduced, or absent

At the same time, the Wilkes stages of all the patients were recorded based on Wilkes staging system^24^.

Bone marrow changes are defined based on the degree of intensity of the bone marrow as seen on MRI in relation to the standard brain intensity (constant reference), as follows:

*Normal:* the condyle bone marrow shows hyperintensity in relation to the brain intensity.

*Mild:* the BM is of an equal intensity as the brain.

*Moderate:* the BM signals on MRI shows hypo intensity in relation to the standard brain intensity.

*Severe:* Condyle BM shows no intensity at all.

### Treatment protocols for ADD patients

According to our staging system, patients in stage 0, stage 1, stage 2 and stage 3A were suggested to undergo disc reposition treatments, including functional splint therapy and arthroscopic or open surgeries. As for patients in stage 3B, MRI was repeated after 6 to 12 months to determine whether condylar resorption had stopped. Once condylar resorption continued, it was treated as stage 4. In this stage, the condyle was routinely resected and replaced with a costochondral graft (CCG) or total alloplastic TMJ replacement. However all the ADD patients had their right to accept our treatment suggestion or give up the treatment protocols. No matter what selection they chose, All of them were required for regular follow-ups.

### Evaluation of the outcomes of ADD

The outcomes of ADD were evaluated using clinical examination, a questionnaire about jaw function and pain, and imaging studies. In the present study, we only focused on the imaging of the TMJ. As for disc repositioning or no treatment patients, MRI was requested regularly. The following criteria were used to define the outcomes:

1. Excellent: There is normal disc position, with condylar regeneration.
2. Good: There is normal disc position with little or no condylar regeneration, but with no progression of resorption for disc repositioning patients; or there is no progression of condylar resorption for patients with no treatment.
3. Poor: There is recurrent disc displacement and/or progression of condylar resorption for disc repositioning patients; or condylar resorption continues for patients with no treatment.

As for condylar reconstruction cases, we focused on CT and panoramic radiographs. MRI was performed occasionally. The following criteria were used to define the outcomes:

1. Good: The graft is well incorporated, with little or no resorption.
2. Poor: There is resorption of the graft.

In the present study, excellent and good outcomes were considered as ameliorative, while poor was deemed as deteriorative.

### Statistics analysis

Statistical analysis was performed with R version 3.3.2. The risk of deterioration was estimated with Cox proportional hazard regression modeling and patients without deterioration were censored at the time of their last available follow-up. The initial assessment was made using the univariate analysis, with continuous variables evaluated as non-transformed, log-transformed and best-fit fractional polynomial transformations. Transformation did not significantly improve the model fit for any variable, so non-transformed data were used. Multivariate model development involved covariate assessment by statistical significance and biological/clinical importance^38^. Variables and interaction terms were entered into multivariate models if the univariate P value was <0.05 or of biological/clinical importance. For each added covariate, the likelihood-ratio (LR) test was used to evaluate the effect of removal on model fit. Covariates were included in the model if P < 0.05, the LR test indicated a significant improvement to model fit (P < 0.05) and/or there was an evidence of biological/clinical relevance. Model discrimination was assessed using Harrell’s concordance index (c-index)^39^, which is similar to an area under the receiver operating characteristic curve but is suitable for the censored data. For the C-index, a value of 0.5 indicates no discrimination while 1.0 indicates perfect discrimination. For the final model, the C-index and 95% confidence interval (CI) were calculated with bootstrap resampling (100 replications) to reduce overfit bias. Model calibration was assessed with plots of the predicted versus observed probabilities of deterioration, using 100 bootstrap resample. The validation and calibration plots were produced with the R software “rms” package. All P values were two-sided, with P < 0.05 considered as a statistically significant.
